# TAT‐dextran–mediated mitochondrial transfer enhances recovery from models of reperfusion injury in cultured cardiomyocytes

**DOI:** 10.1111/jcmm.15120

**Published:** 2020-03-25

**Authors:** Hideki Maeda, Daisuke Kami, Ryotaro Maeda, Yuki Murata, Jun‐ichiro Jo, Tomoya Kitani, Yasuhiko Tabata, Satoaki Matoba, Satoshi Gojo

**Affiliations:** ^1^ Department of Cardiovascular Medicine Graduate School of Medical Science Kyoto Prefectural University of Medicine Kyoto Japan; ^2^ Department of Regenerative Medicine Graduate School of Medical Science Kyoto Prefectural University of Medicine Kyoto Japan; ^3^ Laboratory of Biomaterials Department of Regeneration Science and Engineering Institute for Frontier Life and Medical Sciences Kyoto University Kyoto Japan

**Keywords:** cardiomyocytes, ischaemia reperfusion injury, mitochondrial transfer, oxidative stress, transactivator of transcription

## Abstract

Acute myocardial infarction is a leading cause of death among single organ diseases. Despite successful reperfusion therapy, ischaemia reperfusion injury (IRI) can induce oxidative stress (OS), cardiomyocyte apoptosis, autophagy and release of inflammatory cytokines, resulting in increased infarct size. In IRI, mitochondrial dysfunction is a key factor, which involves the production of reactive oxygen species, activation of inflammatory signalling cascades or innate immune responses, and apoptosis. Therefore, intercellular mitochondrial transfer could be considered as a promising treatment strategy for ischaemic heart disease. However, low transfer efficiency is a challenge in clinical settings. We previously reported uptake of isolated exogenous mitochondria into cultured cells through co‐incubation, mediated by macropinocytosis. Here, we report the use of transactivator of transcription dextran complexes (TAT‐dextran) to enhance cellular uptake of exogenous mitochondria and improve the protective effect of mitochondrial replenishment in neonatal rat cardiomyocytes (NRCMs) against OS. TAT‐dextran–modified mitochondria (TAT‐Mito) showed a significantly higher level of cellular uptake. Mitochondrial transfer into NRCMs resulted in anti‐apoptotic capability and prevented the suppression of oxidative phosphorylation in mitochondria after OS. Furthermore, TAT‐Mito significantly reduced the apoptotic rates of cardiomyocytes after OS, compared to simple mitochondrial transfer. These results indicate the potential of mitochondrial replenishment therapy in OS‐induced myocardial IRI.

## 1 INTRODUCTION

Prokaryotes can horizontally transfer genetic material, such as drug resistance genes,^1^ while it has long been considered that eukaryotes could only vertically transfer their genetic information. The concept was challenged by the observation that subcellular organelles, such as mitochondria, could intercellularly move through the transportation system, such as neural axons mainly constituted by actin filaments, and these are known as tunnelling nanotubes.^2^ These nanotube structures have been recognized in various in vitro and in vivo conditions by independent researchers.^3^, ^4^, ^5^, ^6^ In organs harvested for transplantation, mitochondria that might be encircled by exosomes in the donor body and resident in the donor organ, which could evoke significant inflammation in the recipient following reperfusion. This might occasionally result in primary graft nonfunction and death during surgery.^7^ Mitochondria possess several mechanisms to protect the host such as activating the innate immune response and inducing apoptosis of recipient cells through cytochrome c, which functions as an electron transporter within the respiratory super‐complex.^8^ Reactive oxygen species (ROS) generated by mitochondrial respiratory chains activate mitochondrial antiviral‐signalling protein (MAVS), relaying the signal to NF‐κB and NACHT, LRR and PYD domain‐containing protein 3 (NLRP3) to release IL‐1β and IL‐18.^9^ Upon mitochondrial membrane depolarization, cardiolipin expressed on the outer mitochondrial membrane reinforces NLRP3 activation.^10^ Leakage of mitochondrial DNA from the mitochondrial matrices is a strong ligand of Toll‐like receptor 9 (TLR9), which stimulates NF‐κB.^11^ Given that mitochondria have potential therapeutic effects in certain disease conditions, persistence of exogenous mitochondria in the vasculature or extracellular spaces might be a risk factor for inflammation. It has been reported that direct injection of isolated mitochondria into a damaged heart supported recovery of organ function.^12^ To mitigate the adverse effects of exogenous mitochondria as an inducer of inflammation, it is necessary to develop a method for effective and rapid uptake by target cells.

We previously reported that isolated mitochondria were taken up by cultured cells through macropinocytosis.^4^ Macropinocytosis could occur stochastically, depending upon mutual densities of cells and materials to be engulfed. One strategy to increase contacts between the cells and the material is through their enrichment in a limited space by centrifugation.^13^ The transfer efficiency of isolated mitochondria in several cell types, including mesenchymal stem cells, human dermal fibroblasts and cancer cell lines, is significantly increased by centrifugation. Although the transfer of mitochondrial content was enhanced with centrifugation, every respiratory functional parameter declined with the mitochondrial content, suggesting that it was not an ideal protocol to transfer exogenous mitochondria into host cells. Magnetomitotransfer using magnetic bead‐labelled mitochondria has been described using a human embryonic fibroblast cell line.^14^ As macropinocytosis is initiated by receptor tyrosine kinase (RTK) activation, which is the intracytoplasmic domain of the epidermal growth factor (EGF) receptor, EGF was utilized to enhance the transfer efficiency of exogenous mitochondria into human osteosarcoma cells, resulting in a significant increase in efficiency.^15^ The cell‐penetrating peptide (CPP) family has been intensively investigated to deliver small chemicals or genetic materials into cells.^16^ Among more than hundreds of CPPs, HIV TAT peptide demonstrated superior transfer characteristics of payloads into cells, such as nucleotides,^17^ proteins 18 and small molecules,^19^ and also enabled crossing the blood brain barrier.^20^ Pep‐1 shows less cytotoxicity among CPP family members, and the mode of action is independent of the endosomal pathway, including macropinocytosis. Pep‐1 with isolated mitochondria enabled the functional recovery of fibroblasts derived from patients with MERRF (myoclonic epilepsy with ragged red fibres) syndrome.^21^ Pep‐1–mediated mitochondrial transfer was applied to osteosarcoma‐derived cybrid with mitochondrial diseases and showed a significant improvement in transfer efficiency.^22^, ^23^ There have been no reports of a mitochondrial transfer protocol to enhance the efficiency in primary differentiated cells, such as cardiomyocytes and neurons. The transfer efficiency could highly depend upon the cell type. To investigate protocols to achieve a high transfer rate, the recipient cells for isolated mitochondrial uptake are required to be of the disease cell type.

The prevalence and mortality as a result of coronary heart disease was 16.5 million in the United States between 2011 and 2014 and 0.37 million in 2015 and is the leading cause of death.^24^ Despite advancements in treatment of acute myocardial infarction (AMI), there is a considerable occurrence of heart failure, even after timely and effective reperfusion and, therefore, there are still opportunities for development of novel therapeutics.^25^ The mortality and morbidity for ST‐segment elevation myocardial infarction (STEMI) is approximately 7% and 22%, respectively, despite myocardial reperfusion treatment by primary percutaneous coronary intervention (pPCI) to reduce acute ischaemic injury and infarct size.^26^ The unsatisfactory outcomes in patients receiving pPCI are mainly attributed to acute myocardial reperfusion injury that generates nearly half of the final myocardial infarct size.^27^ Recently, some molecular targets, such as protein kinase G (PKG), reperfusion injury salvage kinase (RISK) and survivor activating factor enhancement (SAFE) signalling, have been identified by analysing the mechanism of ischaemic preconditioning.^28^ Mitochondria are involved in almost all molecular aspects of reperfusion injury either directly or indirectly. To date, direct pharmacological interventions targeting mitochondria to ameliorate reperfusion injury, and several successful animal experiments for cardio‐protection have been reported.^29^ However, there is still no treatment for reperfusion injury, even in combination or by using multiple interventions at different time‐points.^29^

We suggested that exogenous mitochondrial transfer to cardiomyocytes could relieve oxidative stress (OS). Herein, we demonstrated that mitochondrial transfer could be effectively enhanced using a cell‐penetrating protein TAT. In addition, OS‐induced apoptosis resulting in the detrimental release of ROS which occurs at the onset of reperfusion could be ameliorated through mitochondrial transfer in hydrogen peroxide‐treated rat neonatal cardiomyocytes.^30^

## 2 METHODS

### 2.1 Cell culture

Human uterine endometrial gland‐derived mesenchymal cells (EMCs) were kindly provided by Dr Umezawa.^31^ The H9c2 cardiomyoblasts were obtained from the American Type Culture Collection. Stably expressing DsRed‐Mito or GFP‐Mito cells were generated to visualize the isolated mitochondria, as described previously.^4^ Briefly, pMX retroviral vectors carrying Mito‐DsRed were infected into cells and purified by flow cytometry 1 week after retroviral transfection. The EMCs and H9c2 cells were maintained in Dulbecco's Modified Eagle Medium (Thermo Fisher Scientific, Waltham, MA, USA) supplemented with 10% foetal bovine serum (Thermo Fisher Scientific) and 1% penicillin/streptomycin. The cells were incubated at 37°C in a humidified atmosphere with 5% CO_2_.

### 2.2 Isolation of neonatal rat cardiomyocytes

The experimental procedures and protocols were approved by the Animal Experiment Ethics Committee of the Kyoto Prefectural University of Medicine and were performed in accordance with the US Animal Welfare Act. Neonatal rat cardiomyocytes (NRCMs) were isolated from 1‐day‐old Wistar/ST rats and cultured as previously described.^32^ Briefly, the isolated hearts were digested in 0.2% collagenase type II in three cycles. The enzymatically dissociated ventricular myocytes were pre‐plated twice for 30 minutes each for cardiomyocyte enrichment. The isolated cardiomyocytes were seeded into 6‐well culture plates at a density of 1 × 10^5^ cells per well or 24‐well culture plates at a density of 4 × 10^4^ cells per well and maintained in DMEM‐Ham's F‐12 nutrient mixture (Thermo Fisher Scientific) supplemented with 5% newborn calf serum (Thermo Fisher Scientific) and 1% penicillin/streptomycin (Thermo Fisher Scientific).

### 2.3 In vitro model of oxidative stress

Oxidative stress was induced using hydrogen peroxide (H_2_O_2_). NRCMs were washed twice with PBS and incubated in culture medium containing H_2_O_2_ (Wako, Tokyo, Japan) for 2 hours,^33^ followed by reperfusion with fresh medium for 6 hours. The ideal concentration of H_2_O_2_ was determined to 200 µmol/L, based upon cell death under the exposure of H_2_O_2_ at various concentration (Figure S1).

### 2.4 Isolation of mitochondria

DsRed‐labelled mitochondria were isolated from EMCs or H9c2 cells expressing Mito‐DsRed immediately before use by mechanical disruption of cells, followed by differential centrifugation as described previously.^4^ Briefly, cells were ruptured by 10‐20 strokes using a syringe with 27‐gauge needle in homogenization buffer [HB; 20 mmol/L HEPES‐KOH (pH 7.4), 220 mmol/L mannitol and 70 mmol/L sucrose] containing a protease inhibitor mixture (Sigma‐Aldrich, St. Louis, MO, USA). The homogenate was centrifuged at 400 × *g* for 5 minutes to remove cellular debris, and the supernatant was further centrifuged at 6000 × *g* for 5 minutes to pellet the isolated mitochondria‐enriched fraction. The amount of isolated mitochondria was expressed as protein concentration using the Lowry method (Bio‐Rad). The isolated mitochondria were resuspended in 1 mL of HB for their characterization. First, the hydrodynamic size and surface charge (zeta potential: electrostatic potential generated by the accumulation of ions at the surface of colloidal particles) of isolated mitochondria were determined by dynamic light scattering and electrophoretic light scattering measured using a Zetasizer Nano ZS (Malvern Instruments).^34^ Isolated mitochondria were morphologically examined by electron microscopy to ensure enrichment of healthy mitochondria, based on the our previous report.^4^

### 2.5 Preparation of TAT‐dextran complexes

Transactivator of transcription dextran complexes (TAT‐dextran, TAT peptide sequence: GRKKRRQRRRPPQ) were prepared using the conventional reductive amination method. In brief, 10 mg of dextran (molecular weight 35‐50 kDa; MP Biomedicals, LLC) was oxidized by NaIO_4_ (44 mmol/L, Nacalai Tesque. Inc) in double‐distilled water overnight at room temperature and protected from light. The reactant was applied onto a PD‐10 column (GE Healthcare Bio‐Sciences Corp) and freeze‐dried to obtain the oxidized dextran. Next, oxidized dextran (250 µg) and TAT peptide (1011.5 µg, Thermo Fisher Scientific) were mixed in aqueous solution of sodium bicarbonate (0.1 M, pH 8.0) and gently stirred for 6 hours at room temperature. Subsequently, 2.5 µL of 5 M NaBH_3_CN (in 1 M NaOH solution, Wako) was added to the solution, followed by reaction for 30 minutes at room temperature. Then, monoethanolamine (12.5 µL) was added to stop the reaction and stirred overnight. The reactant was purified using a PD‐10 column to obtain the TAT‐dextran complex. The concentration of TAT‐dextran was measured using the conventional phenol‐sulphonic acid method.^35^

### 2.6 Modification of isolated mitochondria and mitochondrial transfer

To obtain TAT‐dextran–modified mitochondria, 2 µg of isolated mitochondria was incubated with 2, 6 or 10 µg of 1 µg/µL TAT‐dextran solution at room temperature for 5 minutes in HB containing a proteinase inhibitor mixture up to 20 µL, immediately before use. Mitochondrial transfer was performed by co‐incubating isolated mitochondria with the cells, as described previously.^4^ Briefly, 2 µg of isolated mitochondria was added to 2 × 10^4^ cells per well of 24‐well culture plates in 400 µL of culture medium containing 116.6 mg/L CaCl_2_ by reverse method and co‐incubated at 37°C in a humidified atmosphere with 5% CO_2_. In the experimental model of OS, 2 µg of control or modified mitochondria was added to each well after induction of OS.

### 2.7 Flow cytometric analysis

Mitochondrial transfer was confirmed by fluorescence microscopy and fluorescence‐activated cell sorting (FACS) analyses as described previously.^4^ Briefly, after washing the cells extensively with PBS, fluorescent images were captured using an IX71 fluorescence microscope (Olympus, Olympus, Tokyo, Japan) or BIOREVO BZ‐9000 fluorescence microscope (Keyence, Osaka, Japan). The DsRed‐positive cell population was evaluated by FACS analysis using 488 and 561 nm laser lines. Fluorescence intensity data were collected using SH800 (Sony) and analysed using FlowJo software (TreeStar).

### 2.8 Comparison of mitochondrial transfer efficiency between co‐culture and co‐incubation methods

In the co‐culture method, GFP‐Mito EMCs were treated with H_2_O_2_ for 6 hours and washed twice with PBS. Following this, fresh medium was added containing DsRed‐Mito MSCs. The two types of cells, GFP‐Mito EMCs and DsRed‐Mito MSCs, were co‐cultured with each cell number of 5 × 10^5^ in 10‐cm culture dish, whereas, in the co‐incubation method, mitochondria isolated from 5 × 10^5^ cells of DsRed‐Mito MSC were added to 5 × 10^5^ of GFP‐Mito EMCs in a culture dish. Subsequently, 24 hours after co‐culture or co‐incubation, the percentage of MSCs containing DsRed‐Mito was calculated.

### 2.9 Total RNA extraction and quantitative RT‐PCR

Total RNA from NRCMs was extracted using TRIzol (Life Technologies) and a Direct‐zol RNA MiniPrep Kit (Zymo Research) with DNase I, according to the manufacturer's instructions. To perform the qRT‐PCR assay, 100 ng of total RNA was reverse transcribed using the PrimeScript RT Reagent Kit and SYBR Premix Ex Taq (Takara Bio, Shiga, Japan), according to the manufacturer's instructions. qRT‐PCR was performed using a Thermal Cycler Dice Real‐Time System using the default cycling program (Takara Bio). The primers used in this experiment were *Bax* (forward primer: 5′‐GGGTGGTTGCCCTTTTCTACT‐3′ and reverse primer: 5′‐CCCGGAGGAAGTCCAGTGTC‐3′) and *Bcl‐2* (forward primer: 5′‐TGGGATGCCTTTGTGGAACTAT‐3′ and reverse primer: 5′‐AGAGACAGCCAGGAGAAATCAAAC‐3′). The relative gene expression levels of NRCM mRNA were normalized to *Gapdh* expression.

### 2.10 Mitochondrial respiration analysis

Oroboros oxygraphy‐2k (OROBOROS INSTRUMENTS) was used to measure cellular bioenergetic changes in cells.^36^ In brief, cells were harvested in 5 × 10^5^/2 mL culture media. The cell suspension was transferred to wells in Oroboros oxygraphy‐2k. After baseline measurements, oligomycin (2 µg/mL), carbonyl cyanide‐p‐trifluoromethoxyphenylhydrazone (FCCP, 1 µmol/L), rotenone (0.5 µmol/L) and antimycin A (2.5 µmol/L) were sequentially added to each well. Data were expressed as the oxygen consumption rates (O_2_ flow per cell; pmol/s/cell). Parameters such as Routine, Basal respiration, ETS, Free Routine activity, ROX, Proton Leakage and Routine coupling efficiency were calculated. For TAT‐dextran–coated mitochondria, respiratory function was measured in MiR05 medium with serially addition of the following compounds; pyruvate‐malate, ADP and oligomycin.^37^

### 2.11 Terminal Deoxynucleotidyl transferase‐mediated dUTP Nick end Labelling Staining

Cardiomyocytes were fixed with 4% paraformaldehyde at room temperature for 20 min and permeabilized with 0.1% Triton X‐100 in 0.1% sodium citrate solution. Apoptotic cells were detected by the Terminal Deoxynucleotidyl transferase‐mediated dUTP Nick end Labelling (TUNEL) method using the In Situ Cell Death Detection Kit, Fluorescein (Roche Applied Science), according to the manufacturer's instructions. Immunofluorescence images were visualized and recorded at 25 random fields per sample using the BIOREVO BZ‐9000 fluorescence microscope (Keyence). The percentage of apoptotic cells was calculated as the ratio of the number of TUNEL‐positive nuclei to the number of DAPI‐ positive nuclei with or without cardiac Troponin T (cTnT) staining.

### 2.12 Statistical analysis

All data are expressed as mean ± SEM. Statistical significance between two groups was determined by unpaired Student's t test using the Microsoft Office Excel analysis tool and GraphPad Prism 6 software (GraphPad Software, San Diego, CA, USA). Results with a value of *P* < .05 were considered as statistically significant.

## 3 RESULTS

### 3.1 Optimization of TAT‐dextran for mitochondrial transfer

**Figure 1:**
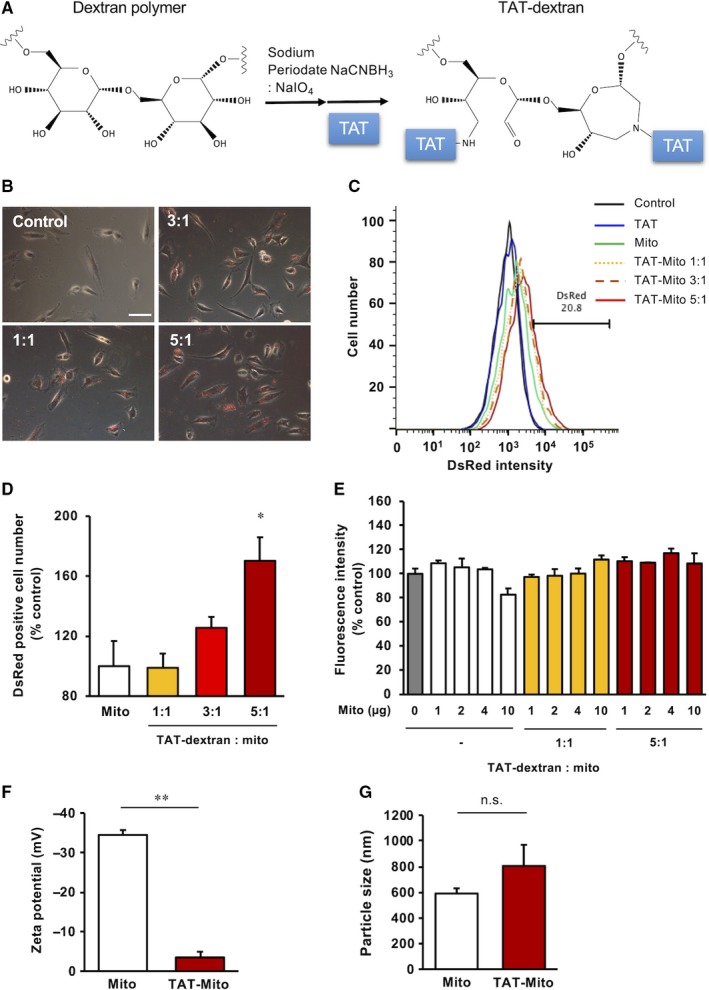
Optimization of TAT‐dextran for mitochondrial transfer. (Recipient cells, EMCs; Mitochondrial donor cells, H9c2 cells). A, Schematic showing the reaction mechanism of TAT‐dextran formation. B, Phase‐contrast microscopy images of EMCs at 24 h after DsRed‐Mito transfer. Ratio indicates TAT‐dextran and mitochondrial concentrations (µg/mL). The white bar indicates 100 µm. C, Evaluation of mitochondrial uptake by EMCs based on DsRed intensity by flow cytometry. D, Graph representing rate of DsRed‐positive EMCs. Error bars indicate standard error (SE) (n = 3). * indicates significant changes compared to mitochondria transfer group (*P* < .05). E, Cytotoxicity analysis of TAT‐dextran with EMCs using MTT assay. Error bars indicate SE (n = 3). F, Zeta potential of isolated mitochondria from H9c2 cells (n = 3). ** indicates significant changes between TAT‐dextran–modified mitochondria and mitochondria without TAT‐dextran modification (*P* < .01). G, Sizes of isolated mitochondria from H9c2 cells (n = 3). n.s. indicates no significant changes

Transactivator of transcription peptides are known as cell‐penetrating peptides and are derived from the human immunodeficiency virus. TAT peptides have an arginine‐rich sequence and have a highly positive charge. Computational analysis of TAT demonstrated stable positive charge (ranging between pH 4 and 10), while Pep‐1 exhibited significant less positive charge that depended on pH, using Protein Calculator v3.4 (http://protcalc.sourceforge.net) (Figure S2A). Conversely, dextran is a water‐soluble, naturally occurring polysaccharide with multiple hydroxyl groups applicable for chemical modification. In this study, the conventional reductive amination method was performed to conjugate TAT peptides to dextran, named as TAT‐dextran (Figure 1A). We previously reported that isolated exogenous DsRed‐labelled mitochondria could be taken up by other cells through co‐incubation.^4^ To increase the efficacy of isolated mitochondrial uptake into cells via co‐incubation, we utilized TAT‐dextran to coat isolated mitochondria (Figure S2B). To determine whether TAT‐dextran adversely affected mitochondrial respiratory function, respirometry was used to examine isolated mitochondria mixed with TAT‐dextran. State3, State4 and the ratio of State3 to State4 were almost the same between isolated mitochondria and TAT‐dextran–coated mitochondria (Figure S3). First, we evaluated the mitochondrial uptake efficacy in H9c2 rat cardiomyoblast cells expressing mitochondria‐targeted DsRed. We modified isolated DsRed‐labelled mitochondria with TAT‐dextran and co‐incubated with the human uterine endometrial gland‐derived mesenchymal cells (EMCs) for 24 hours. TAT‐dextran modification enhanced the internalization of exogenous mitochondria into EMCs in a dose‐dependent manner (Figure 1B). The titration to determine the ratio of TAT‐dextran to mitochondria relative to protein content was performed by FACS analysis, in which a ratio of 5:1 of TAT‐dextran to isolated mitochondria showed the strongest fluorescence intensity of DsRed (Figure 1C). The efficacy of mitochondrial transfer was significantly increased at the ratio of 5:1 as observed by flow cytometric analysis (170.3 ± 15.7%, *P* = .0125 versus Mito group, n = 3) (Figure 1D). Cytotoxicity by TAT‐dextran was not observed even at high concentration (Figure 1E). Next, we examined the efficacy of TAT‐dextran modification on mitochondria isolated from H9c2 cells. The mitochondrial membrane charge and size were measured using a Zetasizer. Isolated mitochondria (Mito) without TAT‐dextran showed a negatively charged surface (−34.5 ± 1.2 mV), whereas isolated mitochondria with TAT‐dextran (TAT‐Mito) showed neutralization of the negatively charged membrane (−3.5 ± 1.4 mV, *P* < .01, n = 3) in a weight ratio of 5:1 for TAT‐dextran versus isolated mitochondria, suggesting that TAT‐dextran fully covered the outer mitochondrial membrane (Figure 1F). Further, the average size of isolated mitochondria was not affected by TAT‐dextran modification (589.5 ± 40.8 nm in Mito versus 804.8 ± 164.4 nm in TAT‐Mito, *P* = .27, n = 3, respectively) (Figure 1G). Therefore, we determined the concentration ratio of TAT‐dextran and mitochondria to be 5:1. It was demonstrated that TAT‐dextran–coated mitochondria fused with endogenous mitochondria in the combination of EMC with genetically GFP‐marked mitochondria as recipient cells and DsRed‐labelled mitochondria at 6 hours after co‐incubation (Figure S4). As isolated mitochondria fused with endogenous mitochondria, it suggested that TAT‐dextran might not inhibit fusions, which might be a mechanism to rescue damaged mitochondria in addition to replenishing respiratory chain complexes. Using the same labelling combination to estimate the amount of exogenous mitochondria replacing endogenous mitochondria in recipient cells, the ratio of DsRed versus GFP was measured (Figure S5A,B). Internalization in TAT‐dextran–coated mitochondria reached to about 30% of the endogenous mitochondria, which was significantly higher than that in isolated mitochondria, based on quantification of fluorescent areas (Figure S5C).

### 3.2 Enhanced cellular uptake of isolated mitochondria into NRCMs by TAT‐dextran

**Figure 2:**
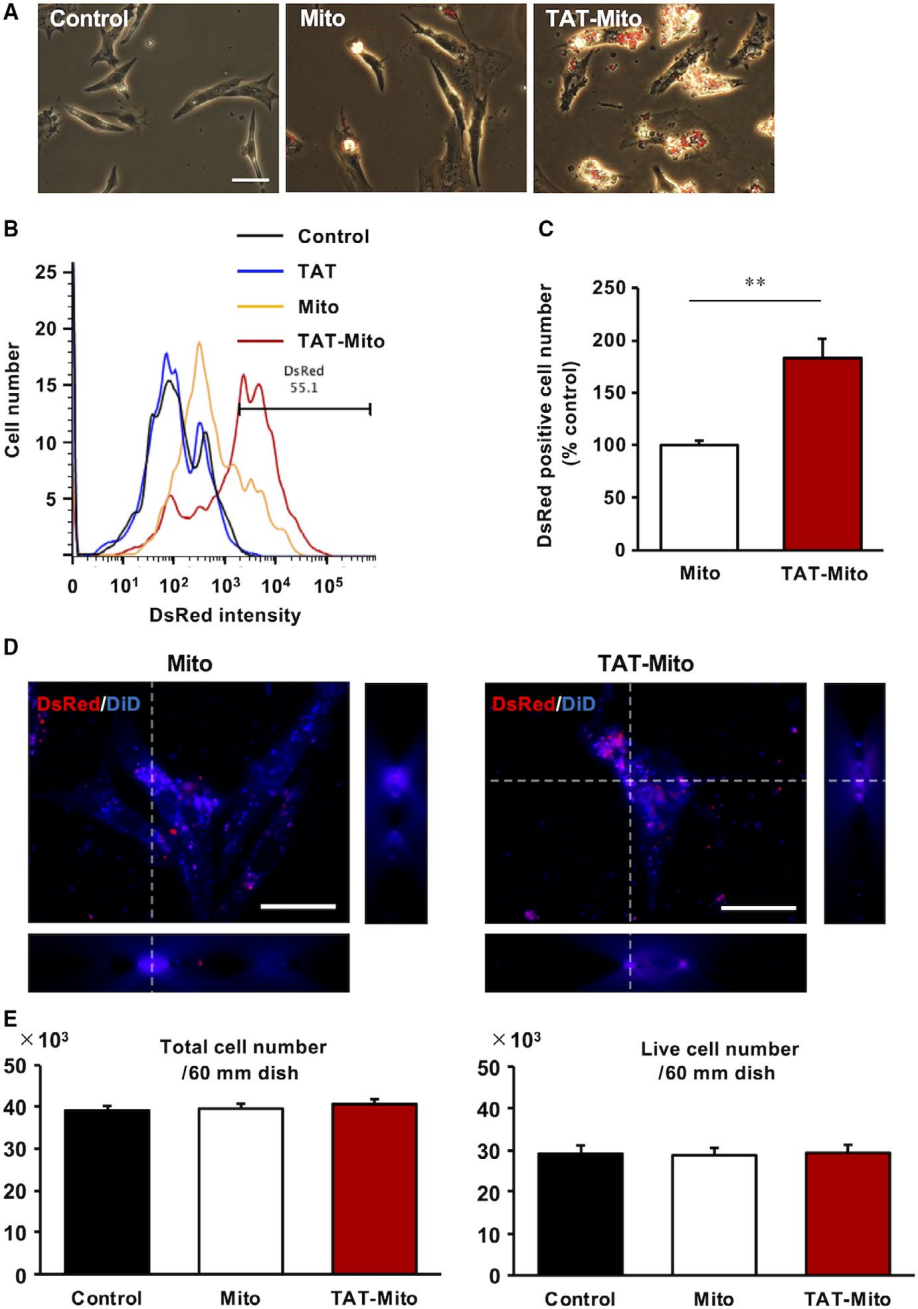
Transactivator of transcription‐dextran enhances cellular uptake of isolated mitochondria in NRCMs. (Recipient cells, NRCMs; Mitochondrial donor cells, H9c2 cells). A, Phase‐contrast microscopy images of NRCMs at 24 h after DsRed‐Mito transfer. The white bar indicates 100 µm. B, Evaluation of mitochondrial uptake by NRCMs based on DsRed intensity by flow cytometry. C, Graph representing rate of DsRed‐positive EMCs. Error bars indicate SE (n = 3). **indicates significant changes compared to mitochondrial transfer group (*P* < .01). D, 3D‐reconstructed images of NRCMs at 24 h after DsRed‐Mito transfer. The white bar indicates 25 µm. E, Cytotoxicity analysis of TAT‐dextran in NRCMs. Error bars indicate SE (n = 3)

We examined whether TAT‐dextran modification of isolated mitochondria enhanced mitochondrial uptake in primary cultured neonatal rat cardiomyocytes (NRCMs). We demonstrated that 24 hours of co‐incubation of NRCMs and DsRed‐labelled isolated mitochondria from EMCs resulted in efficient uptake by using TAT‐dextran (Figure 2A). We confirmed that TAT‐dextran modification significantly increased the internalization of exogenous mitochondria into NRCMs by flow cytometric analysis (182.8 ± 18.4%, *P* < .01 versus Mito group, n = 3) (Figure 2B,C). Additionally, we determined that DsRed2‐labelled mitochondria were localized in the cytoplasm of NRCMs using three‐dimensional reconstructed images. The number of DsRed2‐labelled mitochondria in the cytoplasm seemed to be higher in the TAT‐Mito group of cells than in the Mito group of cells (Figure 2D). Although cytotoxicity is a concern using TAT‐dextran, there were no significant cytotoxic effects observed in NRCMs as a result of TAT‐dextran–modified isolated mitochondria in this study (Figure 2E).

### 3.3 Mitochondrial transfer efficiency is higher in co‐incubation method compared to cell‐to‐cell interaction

**Figure 3:**
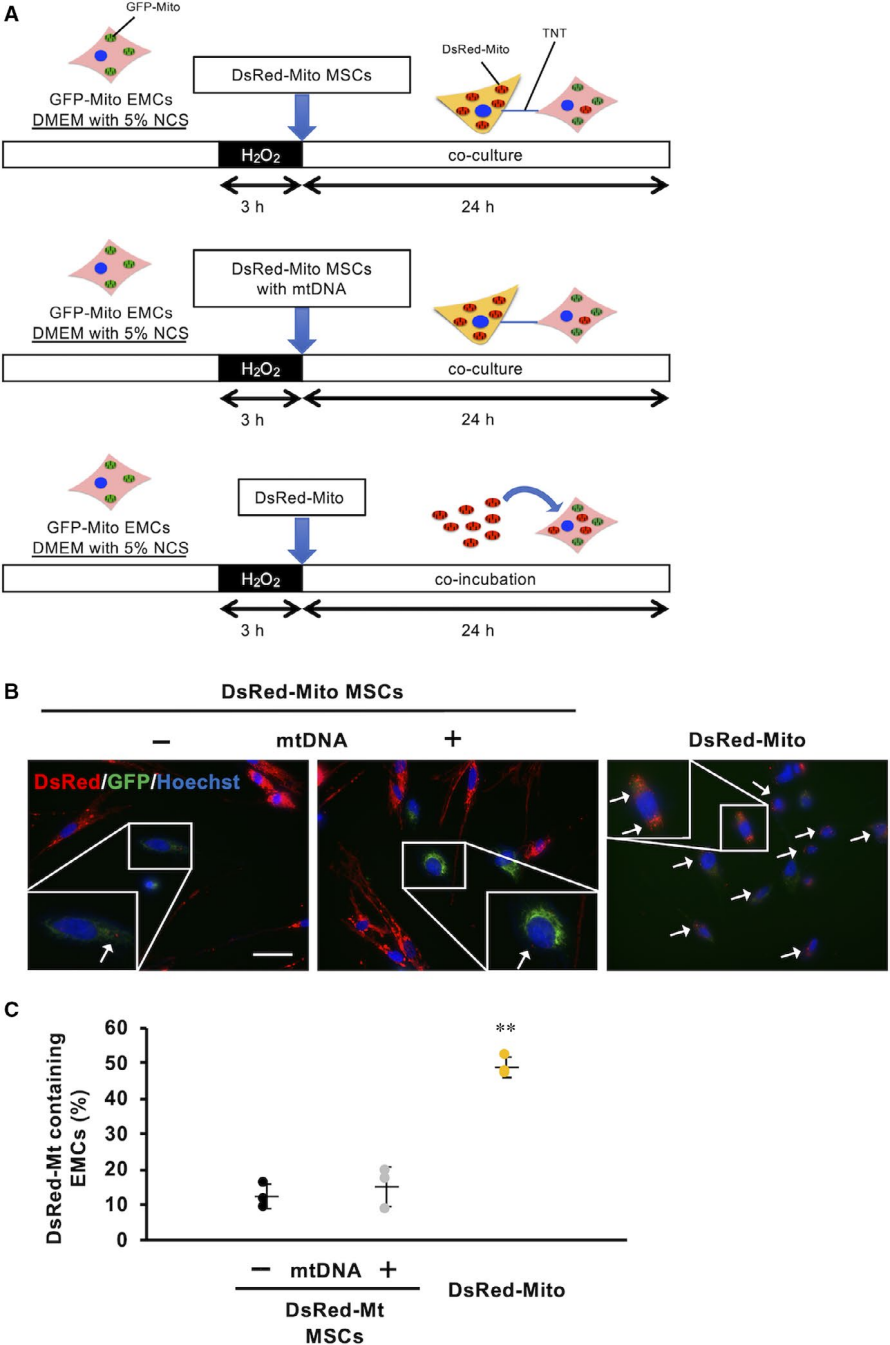
Mitochondrial transfer efficiency is higher in co‐incubation method. (Recipient cells, EMCs; Mitochondrial donor cells, MSCs). A, Schematic representation of each experimental condition. (B, Fluorescent images of EMCs in each condition. White arrows indicate EMCs containing DsRed‐Mito. The white bar indicates 100 µm. C, Evaluation of mitochondrial uptake ratio of EMCs based on DsRed‐positive cells

Tunnelling nanotube (TNT) is known as a bridge to transport intracellular organelles, including mitochondria, to other cells.^2^ Indeed, it has been previously reported that mitochondrial transfer via TNT recovered damaged cells when co‐cultured with healthy cells.^6^, ^38^ Studies have suggested that mitochondrial transfer via TNT was probably induced by damage‐associated molecular pattern molecules (DAMPs) such as mitochondrial DNA (mtDNA).^39^, ^40^ Therefore, to evaluate the efficiency of mitochondrial transfer between co‐culture method using two cell types and co‐incubation of cells with isolated mitochondria, two fluorescent markers GFP and DsRed that are genetically expressed in mitochondria were used for comparison. In co‐culture conditions, we cultured mesenchymal cells (MSCs) that were genetically modified with mitochondria‐targeted DsRed and damaged EMCs that were genetically modified with mitochondria‐targeted GFP in an OS model induced by using H_2_O_2_ 30 (Figure 3A). In a stressed milieu in vivo, various mediators such as DAMPs and an array of pro‐inflammatory cytokines stimulate innate immune responses. We mimicked this situation by adding mitochondrial DNA, a potent inducer of innate immunity, to the culture media. In the co‐culture method, the percentage of EMCs containing DsRed‐mitochondria, which were derived from MSCs, was low (12.4 ± 3.5%) at 24 hours after co‐culture, and the augmented signals of DAMPs upon addition of mtDNA did not affect the translocation of healthy mitochondria (15.1 ± 5.6%) (Figure 3B,C). Simple co‐incubation of isolated DsRed‐mitochondria from MSCs with damaged EMCs for 24 hours demonstrated that DsRed‐Mito content in MSCs was significantly higher (48.9 ± 2.9%, *P* = .0025, n = 3), compared to intercellular mitochondrial transfer (Figure 3B,C).

### 3.4 Bidirectional intracellular mitochondrial transfer and isolated mitochondria concomitantly with DAMPs induce necroptosis‐like cell death

**Figure 4:**
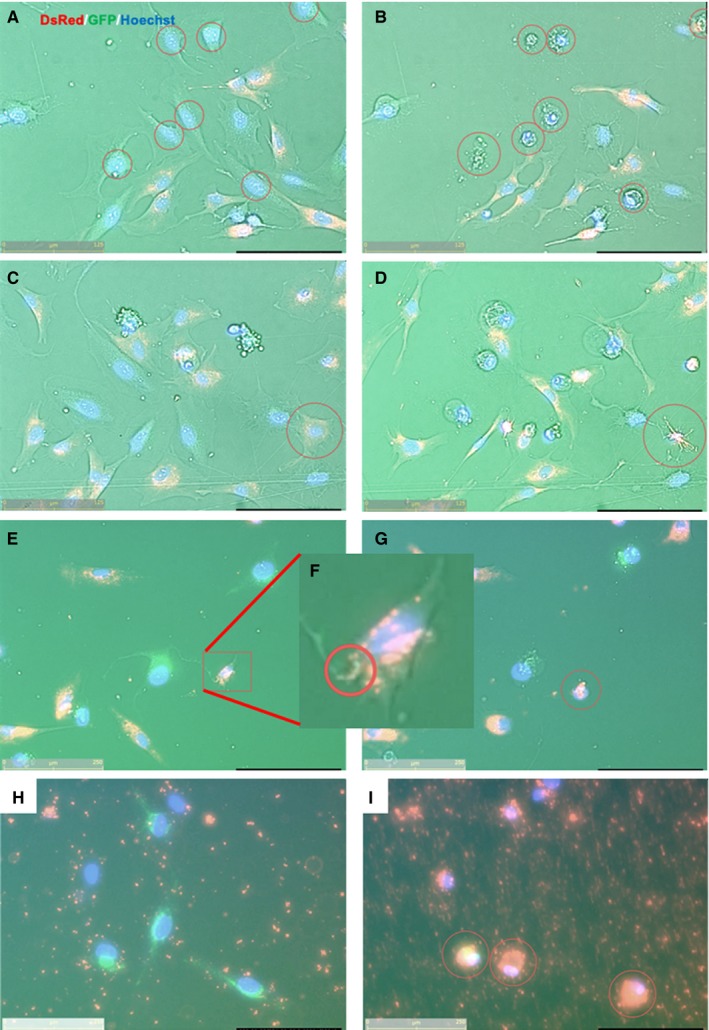
Continuous fluorescence images of mitochondrial transfers in various conditions. (Recipient cells, EMCs; Mitochondrial donor cells, MSCs). (A, C, E and H) Pre‐culture and (B, D, G and I) post‐culture images. (A&B) Damaged EMC cells exhibiting necroptosis‐like cellular bursts at 24 h following 3 h of H_2_O_2_ exposure. (C&D) MSCs which made contact with damaged EMC cells via TNT in the presence of DAMPs were similar in appearance to EMCs as shown in panels A and B, and exhibited necroptosis‐like cellular burst. (E‐G), (F) Enlarged image of E Mitochondrial transfer from damaged EMCs to healthy MSCs via TNT were detected in the presence of DAMPs; consequently, MSCs underwent necroptosis‐like appearance. (H&I) Isolated mitochondria accumulated in damaged EMCs, leading to cell death in the presence of DAMPs. The yellow bar in lower‐left corner indicates 125 µm

Necroptotic cell death mediated via receptor‐interacting protein kinase 3 is a form of regulated necrosis,^41^ which plays an essential role in IRI. In contrast to apoptotic cell death, necroptosis appears as a cellular burst releasing cellular constituents into the extracellular space, which was observed in damaged EMCs either with or without DAMPs (Figure 4A,B, Video S1). Surprisingly, necroptosis‐like cellular bursts of MSCs, which had been initially considered as donor cells of healthy mitochondria, were observed after contact with damaged EMCs with DAMPs (Figure 4C,D, Video S2). We detected that reverse transport of damaged mitochondria from unhealthy EMCs to healthy MSCs with DAMPs via TNT, resulted in a necroptosis‐like appearance (Figure 4E‐G, Video S3). Determination of the directionality of transfer remains to be investigated. MSCs without contact with damaged EMCs did not undergo cellular bursts, suggesting that cell death could be attributed to the reverse transport of damaged mitochondria. However, co‐incubation of isolated mitochondria with recipient cells in the presence of DAMPs exhibited necroptosis‐like cell death (Figure 4H,I, Video S4), suggesting that administration of isolated mitochondria into damaged regions could worsen inflammation and exacerbate organ failure in clinical settings.

### 3.5 Mitochondrial transfer significantly suppresses expression of apoptotic‐related genes in NRCMs after in vitro OS induction

**Figure 5:**
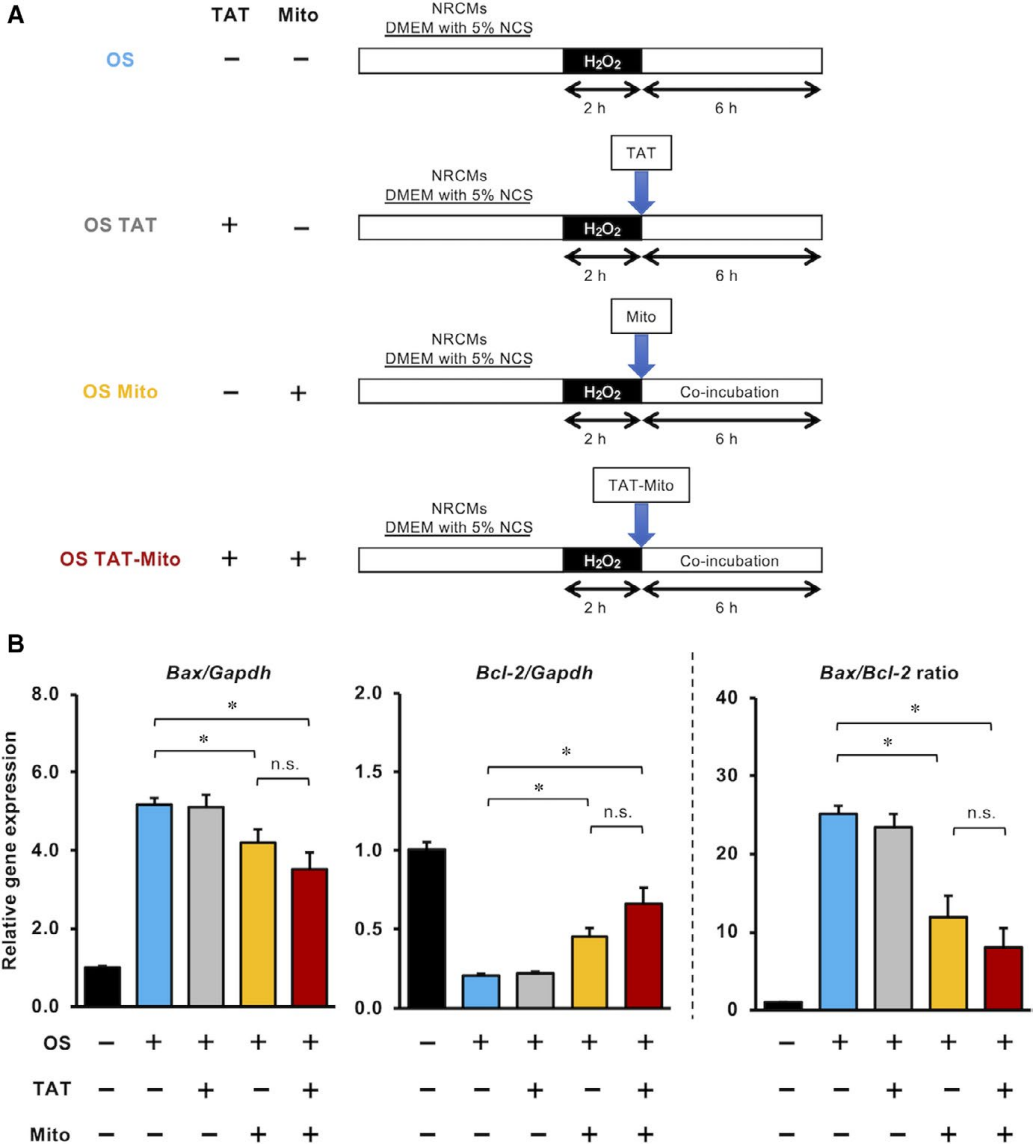
Mitochondrial transfer significantly suppresses the expression of apoptotic‐related genes in NRCMs after in vitro OS induction. (Recipient cells, NRCMs; Mitochondrial donor cells, H9c2 cells). A, Schematic representation of experimental procedure of in vitro OS model in NRCMs. B, Quantitative RT‐PCR analysis of apoptosis‐related gene expression in NRCMs in each condition. The expression levels individual mRNA transcripts were normalized to *Gapdh* expression level. Error bars indicate SE (n = 3). * indicates significant changes compared to untreated controls (*P* < .05). n.s. indicates no significant changes

We examined the effect of TAT‐dextran and isolated mitochondria on apoptosis in an in vitro NRCM model of OS (Figure 5A). After induction of OS, the expression levels of apoptosis‐promoting gene *Bax* in NRCMs was increased approximately five times compared to the control group, while the levels of apoptosis‐suppressing gene *Bcl‐2* was reduced to approximately 25% compared to the control group (Figure 5B). Further, *Bax* and *Bcl‐2* genes were significantly suppressed and promoted, respectively, when co‐incubated with isolated mitochondria (Mito), compared to the OS group (Figure 5B). TAT‐Mito group demonstrated more effective anti‐apoptotic shift, showing a decreased *Bax* to *Bcl‐2* ratio (Figure 5B right).

### 3.6 Mitochondrial transfer might rescue mitochondrial metabolic function in NRCMs after in vitro OS induction

**Figure 6:**
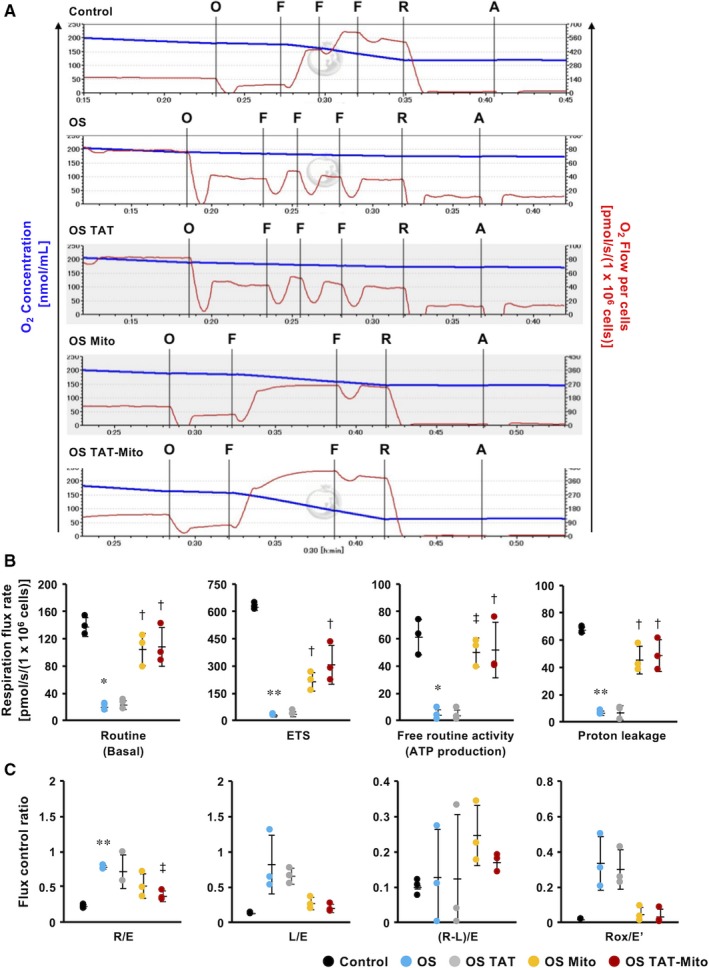
Mitochondrial transfer might rescue mitochondrial function in NRCMs after in vitro OS induction. (Recipient cells, NRCMs; Mitochondrial donor cells, H9c2 cells). A, Coupling control protocol test of NRCMs after in vitro OS induction, showing continuous traces of O_2_ concentration (blue line) and O_2_ consumption (red line). O; Oligomycin (inhibitor of ATP synthase) 2 µg/mL, F; FCCP (potent uncoupler of oxidative phosphorylation in mitochondria) 0.5 µmol/L, R; Rotenone (inhibitor of mitochondrial respiratory complex I) 0.5 µmol/L, A; Antimycin A (inhibitor of mitochondrial respiration) 2.5 µmol/L. B, Rate of respiration flux during each condition. Routine indicates basal mitochondrial O_2_ consumption under endogenous cellular conditions. The electron transport system (ETS) indicates the maximum respiratory capacity of the ETS under each cellular condition. Free routine activity represents the ability of mitochondria to produce ATP via oxidative phosphorylation (OXPHOS). C, Flux control ratio during each condition. R/E; the ratio of routine respiration and ETS capacity. L/E; the ratio of leaked respiration and ETS capacity. (R‐L)/E; phosphorylation‐related respiration (corrected for leaked respiration) as a fraction of ETS capacity. Error bars indicate SE (n = 3). * and ** indicate significant changes compared to control (*P* < .05 and *P* < .01, respectively). † and ‡ indicate significant changes compared to OS group (*P* < .05 and *P* < .01, respectively)

To evaluate the effect of TAT‐dextran along with isolated mitochondria on mitochondrial metabolic function in NRCMs after induction of OS, we measured mitochondrial oxygen consumption using the Oroboros Oxygraph‐2k (Figure 6A). After induction of OS, routine, ETS and free routine activity of NRCMs were significantly reduced compared with the control group (*P* < .05, *P* < .01 and *P* < .05, respectively, n = 3) (Figure 6B). Mito significantly improved routine, ETS and free routine activity compared to the OS group (*P* < .05, *P* < .05 and *P* < .01, respectively, n = 3) (Figure 6B). Specifically, routine and free routine activities in the Mito group showed levels comparable to the control group. Proton leakage in the Mito group also increased significantly, compared with the OS group (*P* < .05, n = 3), which could be due to restoration of ATP production (Figure 6B). TAT‐Mito group also showed similar results as the Mito group. Mito and TAT‐Mito cells recovered from OS‐induced damages; however, they did not show significant changes. Only TAT‐Mito significantly improved R/E compared to the OS group (Figure 6C).

### 3.7 Significant rescue of cardiomyocytes in NRCMs from OS‐induced apoptosis by TAT‐Mito

**Figure 7:**
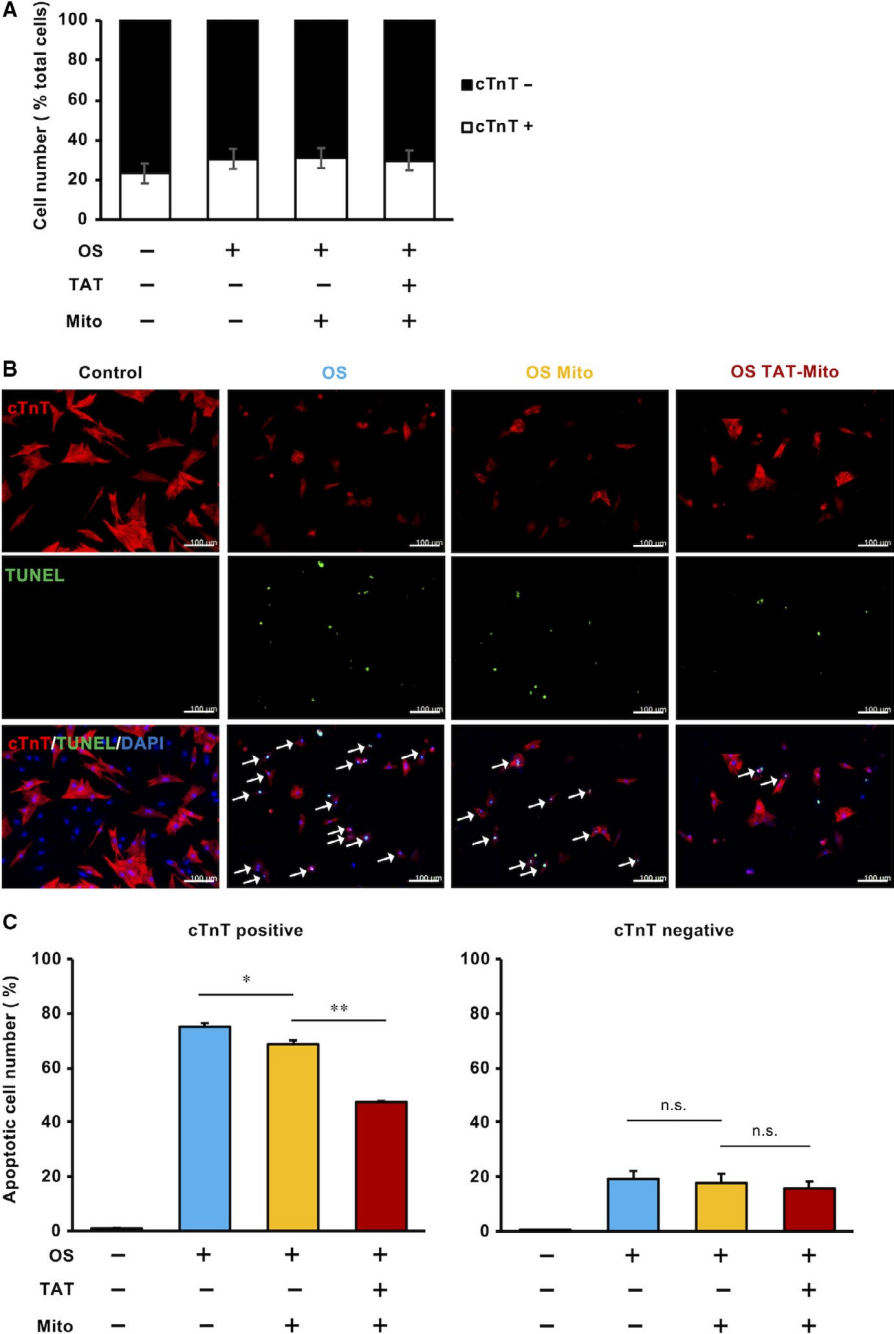
Transactivator of transcription ‐Mito significantly rescues NRCM cardiomyocytes from OS‐induced apoptosis. (Recipient cells, NRCMs; Mitochondrial donor cells, H9c2 cells). A, cTnT‐positive and cTnT‐negative cells in isolated NRCMs. Error bars indicate SE (n = 3). B, Fluorescent images of NRCMs in each condition. White arrows indicate both cTnT‐ and TUNEL‐positive cells. The white bar indicates 100 µm. C, Apoptotic cell ratio of cTnT‐positive (left) and cTnT‐negative (right) cells in NRCMs. Error bars indicate SE (n = 3). * and ** indicate significant changes compared to untreated controls (*P* < .05 and .01, respectively). n.s. indicates no significant changes

Transactivator of transcription‐dextran with isolated mitochondria did not significantly enhance the anti‐apoptotic pathway and metabolic recovery, compared to simple co‐incubation with isolated mitochondria (Figures 5 and 6). We speculated that it could attributed to the mixture of primary cells from rat neonatal hearts. There are various cells such as cardiomyocytes and non‐cardiomyocytes, in NRCMs. There might be differences to OS sensitivity between cardiomyocytes and non‐cardiomyocytes. Therefore, to evaluate the anti‐apoptotic effect of Mito and TAT‐Mito on cardiomyocytes, we analysed these cells separately using a cardiomyocyte‐specific marker, cardiac Troponin T (cTnT). The percentage of cTnT‐positive cells in NRCMs was approximately 30% in all conditions (Figure 7A). The apoptotic rate of cTnT‐positive and cTnT‐negative cells was verified by co‐staining of TUNEL assay and cTnT staining (Figure 7B). After induction of OS, cTnT‐positive cells showed a high apoptotic rate (75.3 ± 1.3%, n = 3) compared to cTnT‐negative cells (19.3 ± 2.8%, n = 3) (Figure 7C). Further, the Mito group showed a significantly reduced apoptotic rate of cTnT‐positive cells compared to the OS group (68.7 ± 2.6%, *P* < .05, n = 3) (Figure 7C). Furthermore, the effect was significantly enhanced in the TAT‐Mito group (47.4 ± 0.5%, *P* < .01 versus Mito group, n = 3) (Figure 7C). However, there were no significant differences in the apoptotic rates of cTnT‐negative cells between each group (Figure 7C). Mitochondrial transfer rescued cTnT‐positive cells in NRCMs from OS‐induced apoptosis. Especially, TAT‐dextran–modified isolated mitochondria prominently enhanced the protective effect of mitochondrial transfer in cardiomyocytes.

## 4 DISCUSSION

Several therapeutic agents and methods that provide myocardial protection have been identified in experimental animal models of myocardial IRI (including NO donors,^42^ H_2_S donors,^43^ metoprolol,^44^ hypothermia 45 and remote ischaemic conditioning 46). However, these therapies have not led to an effective cardioprotective effect in clinical settings.^27^ Targeting one mechanism at a time might be complemented and circumvented, thus limiting the effects of the pathological processes.^29^ These different mechanisms are cooperatively involved in myocardial IRI with temporal variability. Moreover, in addition to cardiomyocytes, myofibroblasts and vascular endothelial cells play different roles in IRI, and each cell type has a different resistance to ischaemia and reperfusion.^47^ Indeed, in our study, cTnT‐positive and cTnT‐negative cells showed different apoptotic rates after in vitro IRI (Figure 7C). To counteract these diverse mechanisms, clinical studies are currently under way to determine whether combined and multitargeted therapies could effectively provide myocardial protection.^28^, ^29^ Mitochondrial transfer alone could have multifactorial cardioprotective effects; therefore, it could be considered as a straightforward method to treat IRI. Exogenous mitochondria could help pleural machinery involved in IRI such as intact respiratory chain complex, to generate energy carriers rather than ROS, and mitochondrial dynamics protein to maintain morphology and segregate pro‐apoptotic materials.

Mitochondria play roles as not only energy generators, but also regulators of apoptosis and hubs for innate immunity.^48^ A key mediator of the intrinsic apoptotic pathway is cytochrome c, which is released from the intermembrane space via aggregates of effector Bcl2 family proteins, such as Bax on the outer mitochondrial membrane (OMM) and Bak recruited in OMM upon apoptosis‐inducing signals. Conversely, anti‐apoptotic Bcl2 family proteins, such as Bcl2 and BclX_L_, and the effector Bcl2 family proteins inhibit apoptosis. Effector and anti‐apoptotic Bcl2 family proteins mutually suppress their function. Fusions between damaged endogenous mitochondria and engulfed healthy exogenous mitochondria might enable anti‐apoptotic Bcl2 family proteins to suppress effector Bcl2 family proteins in damaged endogenous mitochondria, resulting in reduced cytochrome c release and consequently suppressed apoptosis. In addition, rescue of damaged mitochondria by fusions with exogenous healthy mitochondria might suppress intracellular disseminations of mitochondrial DNAs, *N*‐folmyl peptides and mitochondrial transcription factor A, which are strong stimulators of innate immunity and release of pro‐inflammatory cytokines and type I interferons.^11^ In addition, IRI seriously affects Ca^2+^ flux,^49^ which is an essential mediator of mitochondrial metabolism and cell death.^50^ This intervention to mitochondria, providing increments of mitochondrial components, might buffer increased Ca flux from the endoplasmic reticulum into mitochondria under OS. Because this strategy does not replace damaged endogenous mitochondria, cell death could not be completely restrained. However, decreased respiratory function could be compensated through additional respiratory chain complexes with high transfer efficacy.

Although it has been reported that intercellular mitochondrial transport via tunnelling nanotubes (TNT) and exosomes could rescue the damages in several disease models,^6^, ^38^ our study suggested that direct mitochondrial transfer might be more efficient for cellular uptake (Figure 3C) and that TAT‐dextran modification significantly enhanced mitochondrial transfer (Figure 2A‐C). There is a recent report demonstrating the cardioprotective effects of intercellular mitochondrial transfer against IRI in an in vitro model.^51^ However, the directionality of transport of contents via TNT might be context‐dependent and currently cannot be regulated. In some cases, the reverse transport from damaged cells to stress‐tolerant cells induced necroptosis‐like cellular burst (Videos [Link], [Link], [Link], [Link]), which could worsen the inflammatory milieu in in vivo situations. During conditions of stress, mitochondria induce innate immunity by releasing various mediators, like arsenals, such as formyl peptides, cardiolipins, mitochondrial DNA and cytochrome c.^52^ The ejection of damaged mitochondria from surviving cells or donor cells during cell transplantation to attenuate IRI could exacerbate and increase the damage. In this respect, direct mitochondrial transfer might circumvent unpredictable adverse events by exogenous mitochondria with good quality control. Moreover, it could be more effective under stress conditions rather than physiological conditions, as mitochondria are taken up into cells via macropinocytosis which is activated in a nutrient‐deficient milieu.^53^ We need to further investigate the method of mitochondrial transfer to ensure mitochondrial translocation into stressed cardiomyocytes in in vivo conditions. This study provides a premise to investigate the strategy of direct mitochondrial transfer for IRI to in an in vivo setting.

Cell entry is the first hurdle for viral growth. Viruses take advantage of macropinocytosis and receptor‐mediated endocytosis of eukaryotic cells to engulf extracellular materials and fluids.^54^ The machinery that viruses employ to breach cellular membranes has been extensively investigated to develop treatments for diseases by delivering macromolecules intracellularly in conjunction with cell‐penetrating peptides (CPP) derived from human immunodeficiency virus (HIV)^55^, ^56^ and herpes simplex virus (HSV).^57^ HIV TAT mainly utilizes macropinocytosis, although other machineries also function, depending upon cell types, nutritional or redox status of the host, and payloads.^58^ Previously, it has been reported that isolated mitochondria translocate across the cell membrane by macropinocytosis with limited efficiency.^4^ As higher transfer efficiency of isolated mitochondria into cells is required to affect pathophysiological conditions, we initially utilized HIV TAT peptide to augment cellular engulfment. As it is difficult to attach TAT peptides onto mitochondria as a result of electrophysical properties, we generated TAT conjugated with dextran, named as TAT‐dextran in this study. TAT‐dextran possesses suitable properties to neutralize the surface charge of the outer mitochondrial membrane, thereby making contact with the cytoplasmic membrane with the same electric charge. TAT‐dextran successfully demonstrated delivery of isolated mitochondria into primary cardiomyocytes with high efficiency that were not permissive for mitochondrial transfer by simple co‐incubation. Recently, it was reported that a polymer conjugate composed of dextran with triphenylphophonium (TPP) enhanced isolated mitochondria transfer into primary adult mouse cardiomyocytes.^37^ Consistent with the previous study, this study showed that TAT‐dextran maintained the respiratory function of isolated mitochondria and effectively energized damaged cardiomyocytes, suggesting that coated mitochondria might be protected under OS. While TPP is utilized to target mitochondria, TAT facilitates transfer across cell membranes. However, when mitochondria are the cargo, it might not be necessary to direct it to mitochondria; they can cross over the cell membrane instead.

This study demonstrated that efficient mitochondrial transfer was feasible in primary cardiomyocytes and exogenous mitochondria could function so as to alter the physiology of recipient cells. Although studies in cell lines have reported that direct mitochondrial transfer even with poor transfer efficiency has the potential to rescue damaged cells, the methodology described in this study to transfer mitochondria into primary cells with high efficacy could provide the basis for animal studies, and probably for clinical evaluation. In conclusion, our data support direct transfer of TAT‐Mito as a promising approach for the treatment of IRI.

## CONFLICT OF INTEREST

The authors declare no competing interests.

## AUTHOR CONTRIBUTIONS

HM, DK, RM, YM, JJ, TK and SG designed and performed the experiments. HM analysed and interpreted the data. HM, DK and SG prepared the manuscript. SM, YT and SG provided technical support, discussion and reviewed the manuscript.

## Supporting information

Fig S1

Fig S2

Fig S3

Fig S4

Fig S5

Video S1

Video S2

Video S3

Video S4

## Data Availability

The authors confirm that the data supporting the findings of this study are available within the article and its supplementary materials.
