# Supplementary figures and images for: TAT‐dextran–mediated mitochondrial transfer enhances recovery from models of reperfusion injury in cultured cardiomyocytes
Source: J Cell Mol Med. 2020 Mar 25;24(9):5007–20. doi: 10.1111/jcmm.15120 (PMC7205789; doi:10.1111/jcmm.15120)

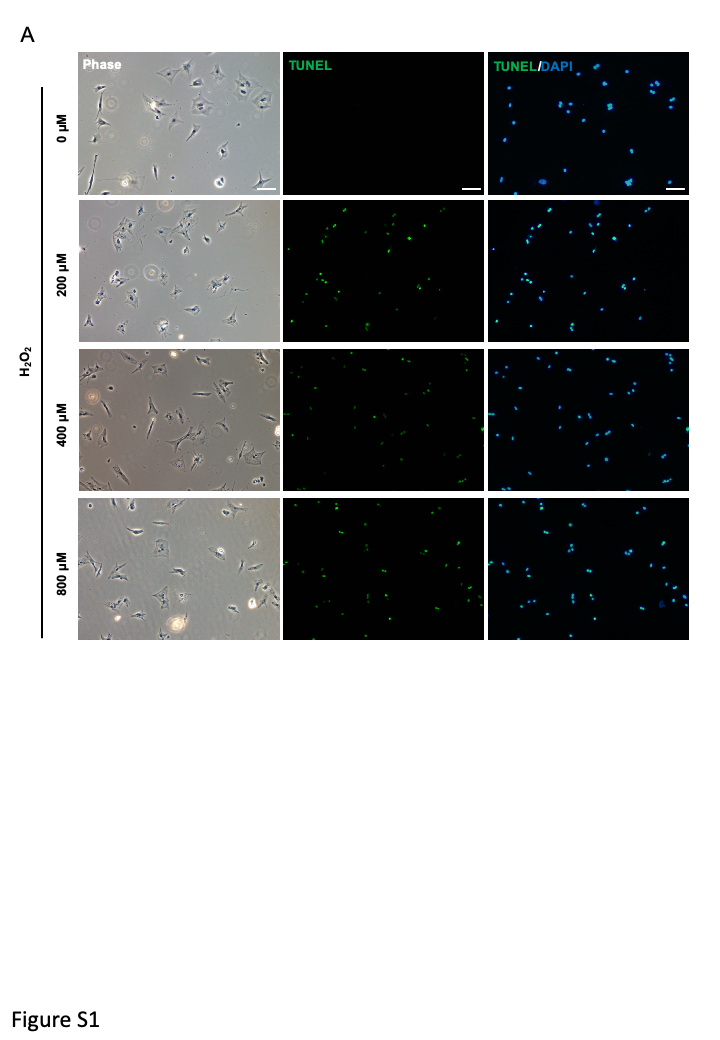

Supplement: Supplementary file 1 — Fig S1 [file JCMM-24-5007-s001.tiff]

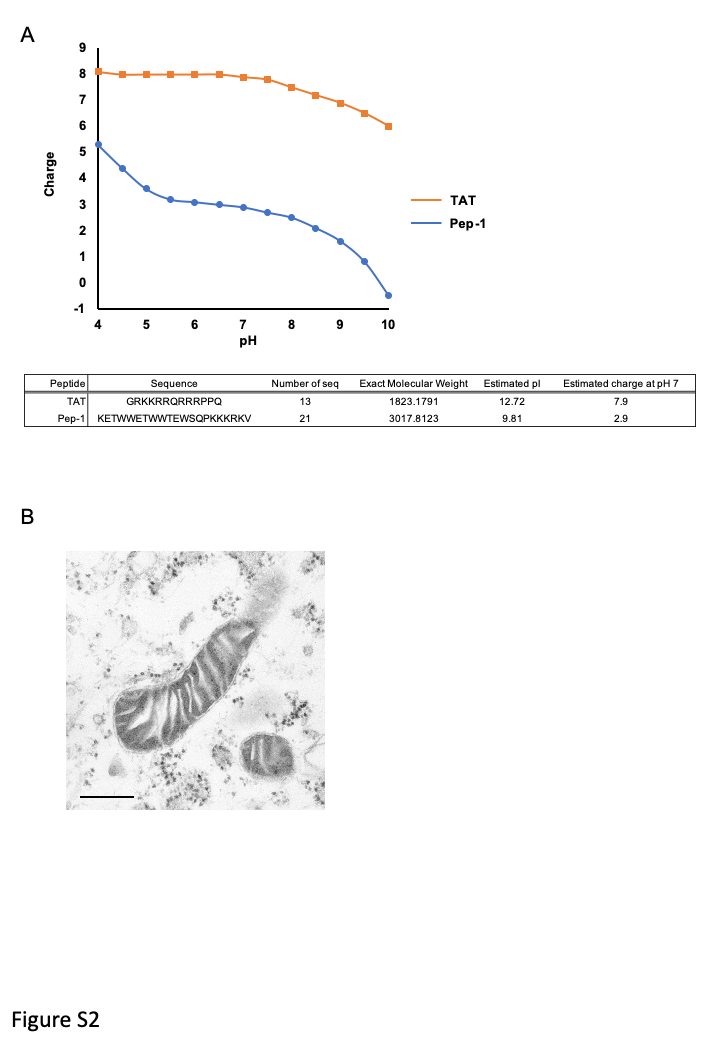

Supplement: Supplementary file 2 — Fig S2 [file JCMM-24-5007-s002.tiff]

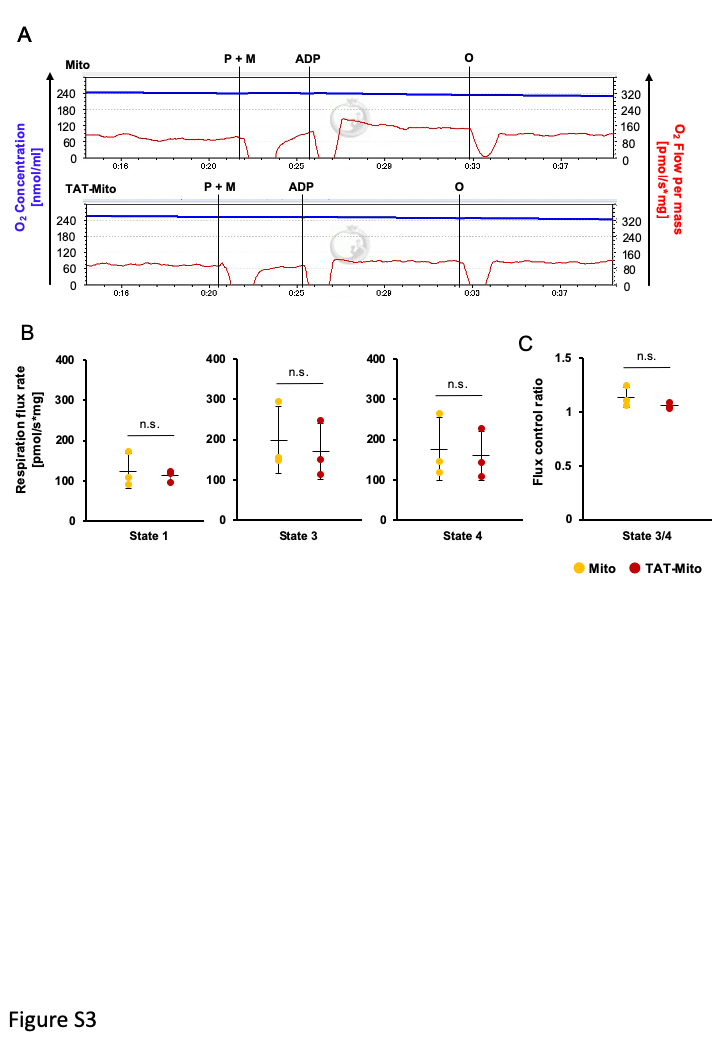

Supplement: Supplementary file 3 — Fig S3 [file JCMM-24-5007-s003.tiff]

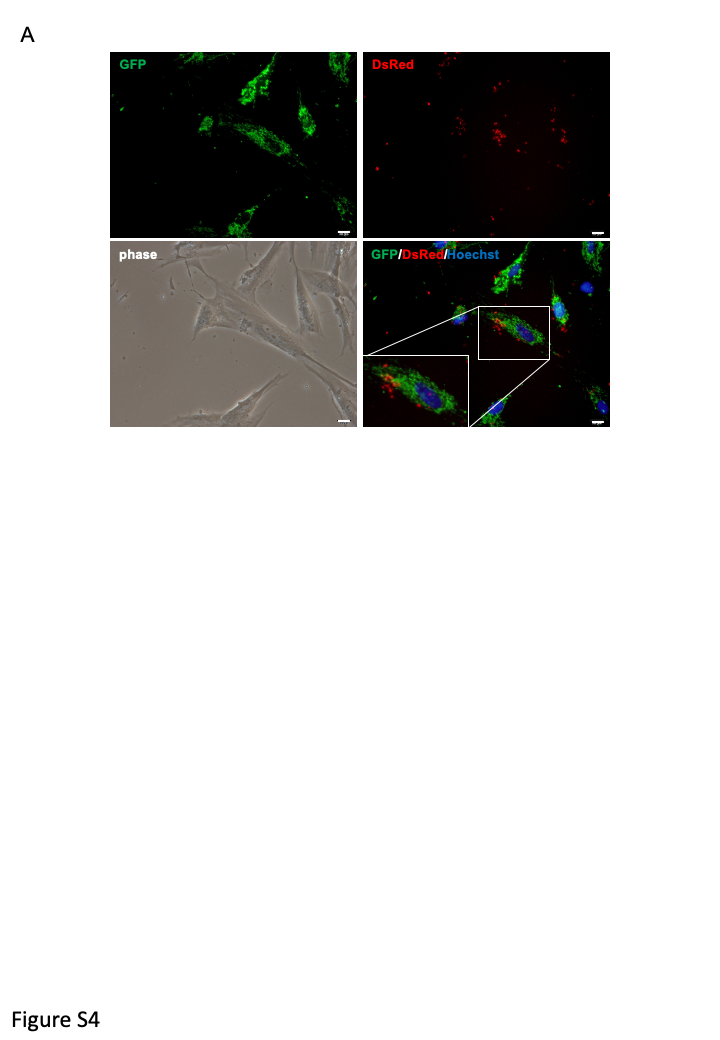

Supplement: Supplementary file 4 — Fig S4 [file JCMM-24-5007-s004.tiff]

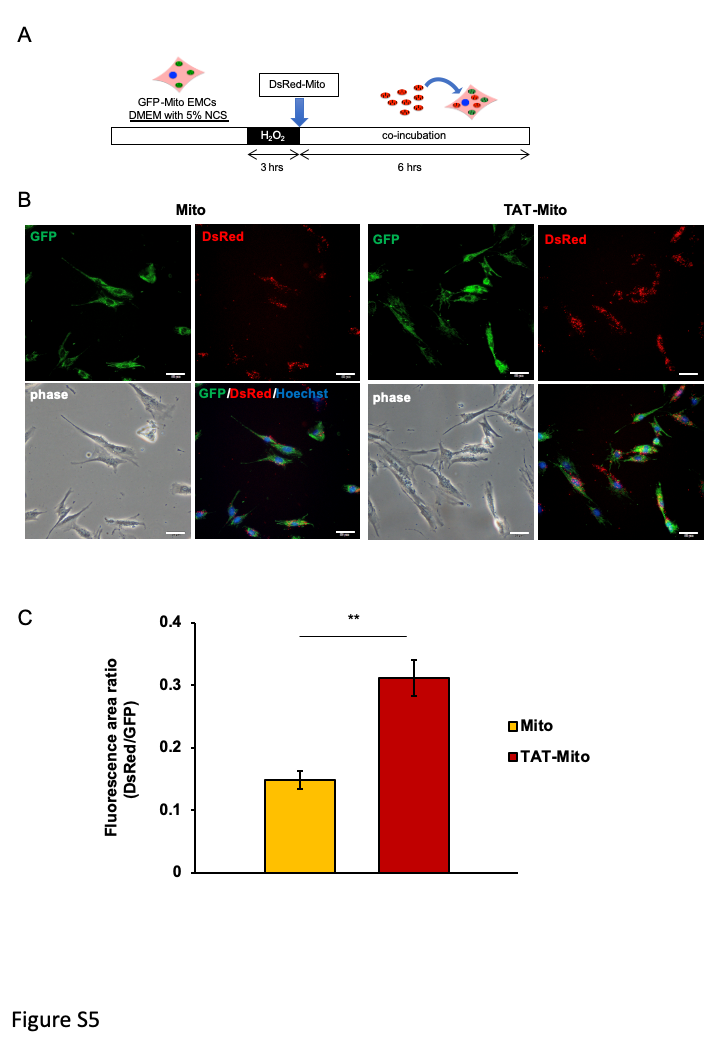

Supplement: Supplementary file 5 — Fig S5 [file JCMM-24-5007-s005.tiff]
